# Management of Primary Brainstem Hemorrhage: A Review of Outcome Prediction, Surgical Treatment, and Animal Model

**DOI:** 10.1155/2022/4293590

**Published:** 2022-07-12

**Authors:** Peng Chen, Haijun Yao, Xiaoyong Tang, Yanglingxi Wang, Qingtao Zhang, Yang Liu, Jin Hu, Yongbing Deng

**Affiliations:** ^1^Department of Neurosurgery, Chongqing Emergency Medical Center, Chongqing University Central Hospital, Chongqing, China; ^2^Department of Neurosurgery & Neurocritical Care, Hua Shan Hospital Affiliated to Fudan University, Shanghai, China

## Abstract

Primary brainstem hemorrhage (PBH) has the worst prognosis of all types of intracerebral hemorrhage. Currently, the management of PBH is controversial. Hematoma classification, scoring systems, and electrophysiological monitoring are critical for predicting the outcome of PBH. Surgery may be an effective treatment for PBH. Clinical studies have emphasized the importance of animal models for understanding the pathogenesis and pathological mechanisms of PBH. In this study, combined with recent studies, the outcome prediction, surgical treatment, and animal models of PBH were reviewed.

## 1. Introduction

The brainstem is located deep in the skull and is small in size and includes the midbrain, pons, and medulla oblongata. The brainstem is mainly composed of nerve nuclei, ascending and descending tracts, and reticular structures. The brain stem is the center that regulates vital functions such as breathing, heart rate, blood pressure, and body temperature. Primary brainstem hemorrhage (PBH) is a spontaneous brainstem hemorrhage associated with hypertension, not associated with cavernous hemangioma, arteriovenous malformation, and other diseases. PBH accounts for 6-10% of intracerebral hemorrhages, with an incidence of approximately 2-4/100,000 per year, with approximately 60-80% of PBH occurring in the pontine region [1]. Its clinical characteristics are sudden onset, rapid neurological deterioration, bleak prognosis, and high mortality (30-90%) [2–5]. PBH most commonly occurs in the age of 40-60 years old, showing trends toward younger age. The incidence is higher in men than in women, and the main risk factor is hypertension; other related factors include anticoagulant therapy, amyloidosis, etc. [1, 5–7].

We reviewed the recent research results of outcome prediction, surgical treatment, and animal model of PBH.

## 2. Outcome Prediction

Since primary brainstem hemorrhage (PBH) has the worst prognosis of all types of ICH, previous studies have investigated prognostic factors such as age, coma, blood glucose, GCS, hemorrhage size, location, and extent of hemorrhage. We will elaborate on prognostic prediction from hematoma classification, scoring system, electrophysiological monitoring, etc.

### 2.1. Hematoma Classification

At present, there is no standard for the classification of PBH in clinical practice. According to the anatomical region, it can be divided into the midbrain type, pontine type, and medulla oblongata type PBH. Medulla oblongata type is the most serious type and may cause ataxic respiration and rapid death [8].

Various classifications were established based on CT images of the anatomical area and direction of expansion of the hematoma. In 1986, Russell et al. classified pontine hemorrhage into three types: central pontine hematoma, dorsolateral tegmental hematoma, and pontine basal hematoma [9]. Based on CT findings, Chung and Park divided pontine hemorrhage into four types: massive, bilateral tegmental, basal-tegmental, and small unilateral tegmental, with survival rates of 7.1%, 14.3%, 26.1%, and 94.1%, respectively [10]. In 2012, Nishizaki et al. combined the previous classification of hematomas with some modifications and classified them into four types, giant, covered to the base, laterally oval, and small unilateral [11]. Wessels et al. reviewed the clinical data of patients with primary pontine hemorrhage and classified into three new types regardless of unilateralism or bilateralism: dorsal, ventral, and massive [5]. It was demonstrated that small amounts of dorsal hemorrhage had a good prognosis, and patients with ventral and massive hematoma has higher mortality [12, 13].

The second affiliated hospital of Zhejiang University School of Medicine in China introduced four types of hematoma classification based on the maximum cross-section of CT and showed the surgical treatment according to different classifications. Type 0 was the hematoma located in the cistern or fourth ventricle, compressed the brainstem, not damage it. Type 1 was the hematoma expanding in half of the brainstem without crossing the midline. Type 2 was the hematoma crossed the midline of the brainstem but does not cross the 3/4 boundary in another side. Type 3 was the hematoma crosses 3/4 of the brainstem. Types 2 and 3 could also be divided into three subtypes including ventral, dorsal, and central [14].

**Figure 1:**
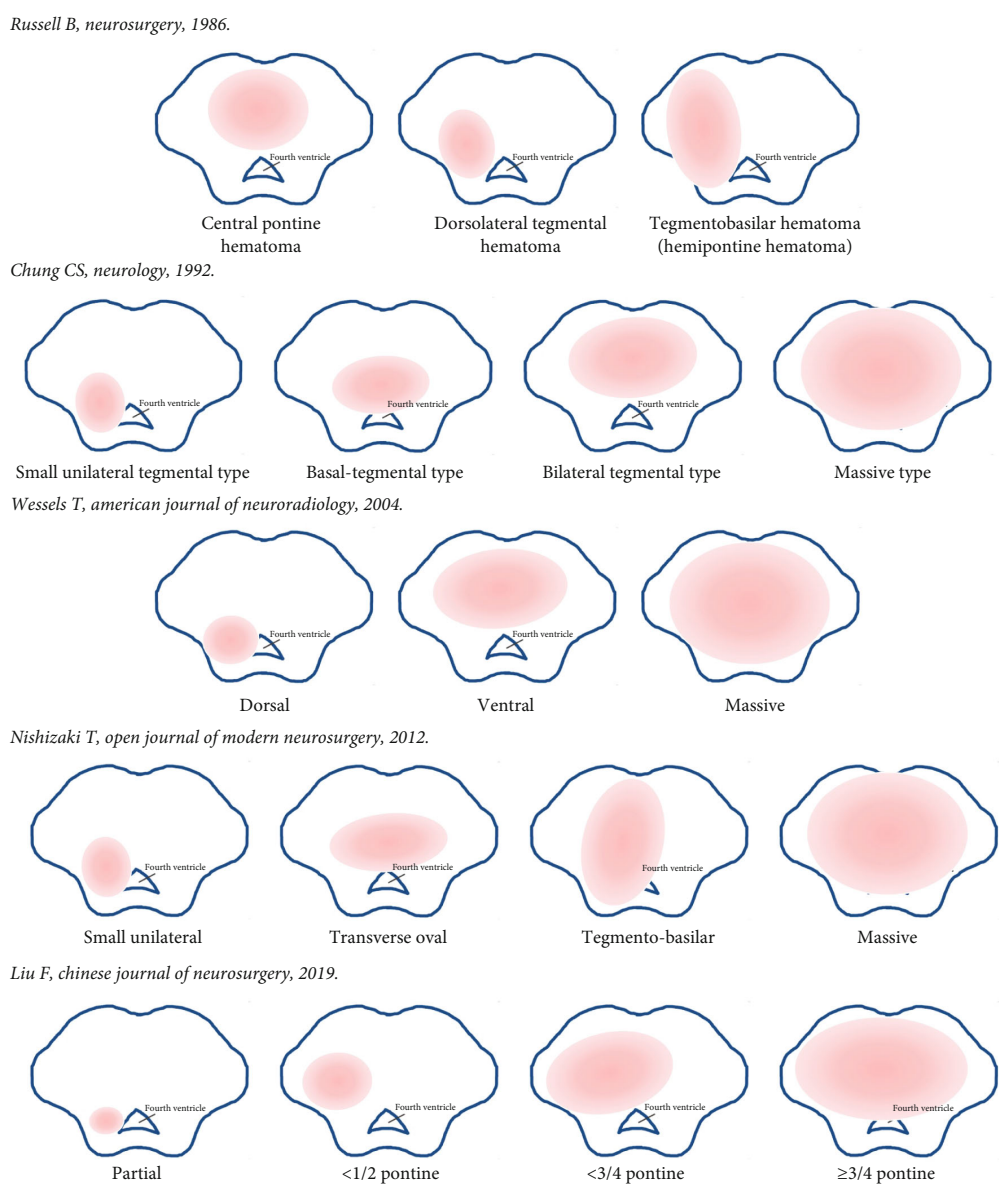
Classification of brainstem hematoma.

Depending on the classification of hematoma, previous studies have shown that the location and volume of the hematoma are most associated with outcome. Unilateral tegmental hematoma has a good prognosis, while bilateral and ventral massive hematomas have the worst prognosis [2]. Classification of brainstem hematoma is presented in Figure 1.

### 2.2. Scoring System

A large number of previous studies have found that predictors of PBH include GCS at admission, location and volume of hematoma, hydrocephalus, disturbance of consciousness, and age. Comprehensive scoring system can play an important role in the management of patients with PBH [12, 15–17].

**Figure 2:**
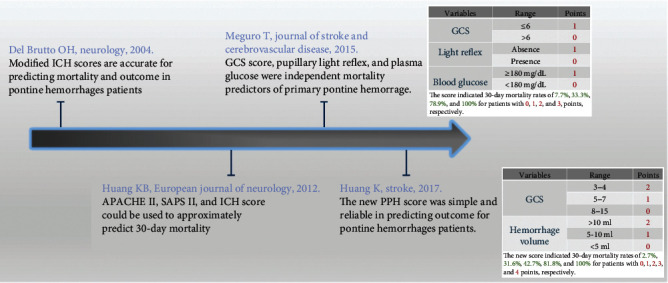
Diagram illustrated development of scoring system of brainstem hemorrhage.

The intracerebral hemorrhage (ICH) scoring has been used to evaluate PBH [18]. Due to the entirely differences of grading of hematoma volume, complex anatomical structures, and blood supply system, the supratentorial ICH scoring system could not be applied to PBH directly and should be modified appropriately [19]. In 2015, Meguro et al. proposed an innovative grading scale (primary pontine hemorrhage) that predicted 30-day mortality of 101 patients with pontine hemorrhage [20]. However, the scoring system, which defined GCS, light reflex, and blood glucose as variables that could influence the search for independent factors, was not externally validated. To address this issue, in 2017, Huang et al. established and validated a comparative scoring system to predict 30-day mortality and 90-day outcome. According to the hematoma volume and GCS score, the hematoma volume is less than 5 mL as 0, 5-10 mL as 1, greater than 10 mL as 2, GCS 8-15 as 0, GCS 5-7 as 1, and GCS 3-4 as 2. The 30-day mortality rates for patients with a total score of 0, 1, 2, 3, and 4 were 2.7%, 31.6%, 42.7%, 81.8%, and 100%, respectively [16]. So far, this was the largest population-based and best evidence score research including 171 patients. The development of scoring system of brainstem hemorrhage is shown in Figure 2.

Significantly, GCS and hematoma volume were the two most influential predictors in scoring system. Future research required abundant samples to establish more accurate scoring system by combining radiographic and laboratory information. Moreover, in addition to the prediction of mortality and functional recovery, the advanced scoring system was responsible in stratification management of therapeutic strategy and surgery indication [21].

### 2.3. Electrophysiological Monitoring

MRI and CT imaging cannot convey complete information on the function of corticospinal tract and sensory pathways. Electrophysiological monitoring was widely used in patients of stroke and was recognized important tool in terms of predicting prognosis after stroke [22]. There was little literature that was available on the utilization of electrophysiological monitoring to predict outcome and guidance therapeutic strategy in patients with PBH.

Electroencephalography (EEG) is widely used for electrophysiological monitoring in clinical practice. Stimulation of the reticular structure always induces EEG changes, and it is difficult to accurately record brainstem functional EEG from scalp monitors alone. Therefore, the application of EEG is mainly in the diagnosis of brainstem death, and the role of EEG in brainstem monitoring remains controversial. Chen et al. found that quantitative EEG variables ((*δ* + *θ*)/(*α* + *β*) ratio, DTABR) were effective in predicting clinical outcomes of 90-day mortality in 31 PBH patients, whereas transcranial Doppler (TCD) was not. It was the first research to verify the importance of quantitative electroencephalogram examination in evaluating prognosis of PBH [23].

Electrophysiological monitoring techniques including somatosensory evoked potentials (SEPs), brainstem auditory evoked potentials (BAEPs), and motor evoked potentials (MEPs) were available to monitor the functional integrity of the pathways passing through the brainstem and provide real-time information to support brainstem surgery [24]. In addition to diagnosing brain death, electrophysiological monitoring parameters have also been considered to be associated with functional recovery after ICH. Chao et al. reported a case with delayed trigeminal motor denervation resulting in developing progressive atrophy of the right temporalis and masseter muscles after pontine hemorrhage, which was consistent with electromyography monitoring results [25]. Kim et al. also reported a case with profound hearing loss on the pure-tone audiogram after pontine intracranial hemorrhage, which was predicted by abnormal brainstem auditory evoked potentials [26]. Seong et al. confirmed that compared with hematoma volume and transverse diameter, combined MEPs and SEPs was a reliable and useful tool for predict outcome of functional recovery (global disability, gait ability, and trunk stability in sitting posture) after primary pontine hemorrhage [27]. The mortality and disability rates of PBH are high, and multimodal electrophysiological monitoring is recommended to explore and analyze the relationship between massive parameters and potential prognosis.

## 3. Surgical Treatment

PBH was critical; therapeutic principles included maintain the stability of vital signs, reduce primary damage, prevent secondary damage, and restore neurological function and structure to the greatest extent. The conservative management strategy of PBH mainly referred to strategy of ICH [28], including management of blood pressure, blood glucose, temperature, coagulopathy and treatment of intracranial hypertension, epilepsy, deep venous thrombosis, and other complications [29–31].

Surgical treatment of PBH is controversial. Brainstem hemorrhage has been excluded from previous trials such as STICH and MISTIE, and there is no clear evidence of the effectiveness of surgery for PBH. The purpose of surgical treatment is to remove or reduce the hematoma, decompress the brainstem, prevent secondary damage, and be sensitive to the brainstem. Next, we will elaborate it from the aspects of surgical perspective, indication, and option.

### 3.1. Surgical Perspective

There were significant differences in the perspective of life between east and west culture, so there were obvious different therapeutic strategies of PBH in different countries. It was generally believed that in European and American countries, severe disability or vegetative state was a high mental and economic burden for patients themselves and their families, and it was difficult to accept the reality of poor prognosis. As a result, western countries hold negative opinion on surgical treatment for brainstem hemorrhage. Moreover, the relevant guidelines did not recommend surgical treatment for brainstem hemorrhage.

In countries represented by China, Japan, and South Korea, the concept of “filial piety” plays an active and unique role in family interactions. Most PBH patients are in a coma, and the treatment strategy is mostly determined by the patient's spouse and children. When families decide on treatment options, Chinese families are more likely to opt for surgery even if they are told the expected outcome is poor. Under this circumstance, Chinese doctors have performed a large number of surgical treatments for PBH and have summarized and accumulated a lot of experience in surgical selection, monitoring methods, complications, and medical assistance.

### 3.2. Surgical Indication

Analysis of outcome prediction for surgical patients with primary pontine hemorrhage, Tao et al. demonstrated that younger age (less than 65y), smaller hematoma (less than 5 mL) without rostrocaudal extension, unilateral hemorrhage, and higher GCS (6–8) could benefit from surgery [21]. The surgical indication was proposed by Shrestha et al. included the mass hematoma volume more than 5 mL, GCS less than 8, and progressive neurological deterioration, especially with extremely unstable basic vital signs on long-term ventilator usage [32]. According to the scoring system for brainstem hemorrhage established by Huang et al., the score of 4 was contraindicated, and patients with the score of 2 to 3 could benefit from surgical treatment. However, based on the current limited evidence, this conclusion would carry out further verification [16]. A review of 10 cohort studies showed that the surgical group included age of 45-65 years, unconscious, GCS was 3 to 8, and the hematoma volume of about 8 mL. It was revealed that elder and comatose in PBH patients were not contraindicated for surgery, and patients in the surgery group required a lower mortality rate and better outcome compared with conservative management [33].

The principles of supratentorial ICH surgery cannot be directly applied to PBH due to differences in anatomy and blood supply systems. In a study of 46 patients with PBH, Lan et al. found that in the early surgery group (≤6 h), 20 patients who underwent surgery had better neurological recovery [34]. However, Chen et al. summarized the experience of 52 cases of PBH in a single center and proposed that the optimal surgical time was 12 to 48 hours by microsurgery [35]. The recommended treatment time of the Chinese clinical nerve repair PBH treatment guidelines is 6-24 hours after bleeding, and the bleeding volume is greater than 5 mL, or the diameter of the hematoma is greater than 2 cm. For patients with blood loss less than 3 mL and no obvious ventricular dilatation or disturbance of consciousness, surgery is not recommended. Surgery is also not recommended for patients with bleeding greater than 15 mL, severe irreversible damage source, and extremely unstable basic vital signs [1].

### 3.3. Surgical Option

Surgical option for PBH included minimally invasive hematoma puncture and drainage, craniotomy hematoma clearance, endoscopic hematoma clearance, etc.

#### 3.3.1. Minimally Invasive Surgery

Takahama et al. have firstly performed the surgery of stereotactic brainstem hematoma puncture and drainage in 1989 [36]. This minimally invasive surgery could reduce the damage of the important structure of the brainstem as far as possible. It has the characteristics of simple operation, short operation time, and minimally invasive craniopuncture combined with urokinase or rtPA infusion. It was found that a slightly lower mortality rate in the hematoma aspiration group than the microscopic surgery group (24.4% versus 31.6%, *P* = 0.162) by Zheng et al. Because of less surgical trauma and shorter operation time, it indicated that hematoma aspiration may be suitable for older patients [33]. The most critical technique for the puncture operation was precision. The common stereotactic methods included the use of frame technology, frameless technology, neuronavigation system, and surgical robot; those greatly improved the accuracy of puncture.

3D-printed technololg has been succeeded applied in medical fields such as intraoperative navigation [37]. Wang et al. applied 3D printing navigation for puncture and drainage of PBH in 2020 and found that the actual puncture target was accurately positioned in the hematoma in 7 cases, and the effect was satisfactory. 3D printing-assisted hematoma puncture and drainage offers an innovative and promising approach for surgical treatment of PBH [38].

At present, mixed reality technology was utilized to construct three-dimensional model of hematoma, puncture trajectory, and anatomical structures with holographic images. Operators could obtain real and virtual image information at the same time by wearing special equipment and interacted with model in the display environment according to their own commands. The technology provided operators with immersive feeling that difficult to fully express through photos or videos or even AR technology. We have performed minimally invasive puncture and drainage in patients with PBH by mixed reality holographic navigation technology and concluded that it was feasible to become an innovative, effective, and safe solution [39].

#### 3.3.2. Craniotomy Surgery

In 1998, Hong et al. in South Korea firstly performed craniotomy hematoma clearance in PBH, and craniotomy hematoma clearance has become an important method for PBH [40]. The advantages of microscopic craniotomy hematoma removal are the maximum hematoma removal under direct vision, clear hemostasis surgery, removal of the fourth ventricle hematoma, and avoidance of cerebrospinal fluid circulation disorders and secondary hydrocephalus. Microsurgery requires high electrophysiological monitoring and surgical skills. Use a microaspirator to absorb the hematoma as much as possible, avoiding damage to normal brainstem tissue and function. Hemostatic gauze should be applied gently to stop bleeding. If bipolar coagulation is performed, it needs to be performed at the same time as flushing water to reduce temperature and thermal damage to the brainstem.

Ichimura performed surgery on 5 patients with mild brainstem hemorrhage, and adopted different surgical positions and approaches according to hematoma location in brainstem. Postoperative consciousness, motor performance, and mRS grades were improvement in all cases [41]. Chen et al. performed neuronavigation-assisted microsurgery on 52 patients with PBH and indicated that in patients with hemorrhage volume less than 10 mL, microsurgery was very rapid, effective, and safe [35]. Previous studies indicated avoid damaging to the brainstem, the craniotomy approach was very important; the craniotomy approach was very important. With the widening knowledge of safe approaches and entry zones for the brainstem, surgical craniotomy, training, and research in brainstem promised to become more precise and attractive [42–44].

#### 3.3.3. Endoscopic Surgery

In 2003, Takimoto et al. firstly introduced a new method that with the aid of neuroendoscopy to evacuate pontine hemorrhage and provide another surgical option [45]. It was challenged to approach ventrally located brainstem hematoma by traditional transcranial approaches. As a result of its development and advantages such as the natural surgical corridor, direct visualization, adequate exposure of structure, and minimal brain or vascular retraction, endoscopic endonasal approach technology may become a feasible alternative to treat ventral brainstem hematoma [46].

Liu et al. and Topczewski et al. reported evacuation of ventral hematoma in the brainstem through endoscopic endonasal approach assisted by intraoperative electrophysiological monitoring, and neuronavigation system achieved good results [47, 48]. However, this procedure required a longer learning curve for surgeons and was lack of high-quality evidence and enough experience to support. Therefore, it was necessary to verify the safety and feasibility of surgery in large clinical samples.

## 4. Animal Model

With low incidence of PBH, it was difficult to perform a large clinical sample research. The plight clinical research emphasized that animal model of PBH was important to understand the pathogenesis and pathological mechanism and provide reference to surgical timing, surgical approaches, etc. At present, the common PBH animal models were mainly constructed by rabbits and rats, and the construction methods were mainly stereotactic injection of collagenase (type VII) or autologous blood.

In 2005, Jin et al. used long-eared Japanese rabbit to construct brainstem hemorrhage model at the level of inferior colliculus by injecting 0.1 mL of its own blood into the brainstem. According to cerebrospinal fluid circulation disorder through hematoma compressing in aqueduct of midbrain, the pressure of lateral cerebral ventricle was increased [49]. It was found that the more PBH model was established in rats gradually. Lekic et al. stereotactically infused 0.15 units of collagenase (type VII) into the ventral pontine tegmentum of the rats to build model. Collagenase was used to simulate the procedure that specifically degrading the collagen components of intercellular matrix and basement membrane of vascular endothelial cells, which was closer to the pathological procedure of spontaneous intracerebral hemorrhage. This new PBH rat model demonstrated neurological deficits and brain edema and was used to test treatment strategies for PBH [50, 51]. Guo et al. also established a PBH model in rats by stereotactic injecting type VII collagenase to observe the pathological procedure of secondary injury caused by iron overload and concluded that administration of deferoxamine had limited therapeutic effects on collagenase-induced brainstem hemorrhage in rats [52, 53].

A rat model was established by stereotaxically injecting 30 *μ*L of autologous whole blood from the central caudal artery at a constant rate of 5 *μ*L/min with a microinfusion pump. Brain edema, white matter damage, and neurological deficits due to stable hematomas in this new model are helpful for future studies of pathophysiological mechanisms and evaluation of new treatments [54]. Recently, Tang et al. provided a protocol to establish a massive pontine hemorrhage model in a rat. Firstly, 100 *μ*L of autologous blood from the tail vein was acquired and stereotaxically injected only 10 *μ*L into the pontine. Secondly, the syringe was left in position for 20 min to prevent blood from flowing into the subarachnoid space. Thirdiy, restart injection at the same speed of 1 *μ*L/min until the residual blood has been injected completely. It was concluded that the symptoms of this model were in line with patients with massive pontine hemorrhage [55]. Shrestha et al. provided a reliable and reproducible model for rat brainstem hemorrhage and summarized key points to success in modeling, such as stereotactic location, blood volume (twice injection, more than 60 *μ*L), anesthesia, head positioning, autologous blood coagulation, needle deviation, brain shift, and back-leakage [56].

## 5. Conclusion

In conclusion, PBH is clinically characterized by low morbidity, high mortality, and poor prognosis. Currently, there are different hematoma classifications for PBH in clinical practice. The results showed that unilateral tegmental hematoma had a good prognosis, while bilateral and ventral massive hematomas had the worst prognosis. GCS and hematoma volume were the two most influential predictors in the scoring system. Multimodal electrophysiological monitoring, such as SEPs and MEPs, is recommended to explore and analyze potential prognoses. Surgical treatment of PBH is controversial. There are significant differences in the perspectives on surgical treatment between Eastern and Western cultures, and there are significant differences in recommended strategies. At present, studies have confirmed that surgical options such as stereotactic hematoma puncture and drainage, craniotomy hematoma removal, and endoscopic hematoma removal can benefit appropriate patients, but high-quality evidence and sufficient empirical support are still lacking. Dilemma clinical studies have emphasized that PBH animal models provide important reference for understanding its pathogenesis and pathological mechanisms, as well as treatment.

## Data Availability

Data was provided in the manuscript.
